# Lymphomas associated with Epstein-Barr virus infection in 2020: Results from a large, unselected case series in France

**DOI:** 10.1016/j.eclinm.2022.101674

**Published:** 2022-10-01

**Authors:** Marie Donzel, Maxime Bonjour, Jean-Damien Combes, Florence Broussais, Pierre Sesques, Alexandra Traverse-Glehen, Catherine de Martel

**Affiliations:** a Hospices Civils de Lyon, Institut de Pathologie Multisite, Hôpital Lyon-Sud, Pierre Bénite, France; b Early Detection, Prevention and Infections Branch, International Agency for Research on Cancer (IARC/WHO), Lyon, France; c Hospices Civils de Lyon, Service d'Hématologie, Hôpital Lyon-Sud, Pierre Bénite, France; d Centre de Recherche en Cancérologie de Lyon; INSERM Unité Mixte de Recherche (UMR)-S1052; Centre National de la Recherche Scientifique UMR 5286, Université Claude Bernard Lyon 1, Lyon, France

**Keywords:** Lymphoma, EBV, Prevalence, Epidemiology, Hodgkin lymphoma, Non-Hodgkin lymphoma

## Abstract

**Background:**

Despite mounting evidence for a causal role in an increasing number of lymphoma subtypes, very few studies have systematically tested the entire spectrum of Hodgkin and non-Hodgkin lymphomas for the presence of Epstein-Barr virus (EBV). Here, we describe the prevalence of EBV in a large, unselected series of patients diagnosed with any type of lymphoma during 2020, in the pathology department of a single University Hospital in France.

**Methods:**

A total of 756 lymphoma cases (89% new diagnoses and 11% relapses), were registered in the department between Jan 1 and Sept 30, 2020 and 616 were successfully tested for EBV presence in tumour cells by EBV-encoding RNA in-situ hybridisation, using double-blinded assessment and a scoring system designed in accordance with the current state of knowledge in the literature

**Findings:**

A strong association with EBV was described in 27/87 (31%) classic Hodgkin lymphomas, 12/223 (5%) diffuse large B-cell lymphomas, and 18/71 (25%) NK and T-cell lymphomas: 4 extranodal NK/T-cell lymphomas, nasal type, 14 angioimmunoblastic T-cell lymphomas (48%). In Hodgkin and NK and T-cell lymphomas, there was a statistically significant association between EBER positivity and relapse (*p* < 0·01). Among other subtypes, a potential association with EBV (≥10% stained cells) was found in 2/97 (2%) follicular lymphomas, both of grades 1–2, 1/19 (5%) chronic lymphocytic leukaemia (CLL), 1/9 lymphoplasmacytic lymphomas (11%), and 2/47 (4%) marginal zone lymphomas.

**Interpretation:**

When applied to the distribution of lymphomas in France as described in the Lymphopath database, our data suggested that at least 8% of all combined Hodgkin and non-Hodgkin lymphomas are associated with EBV.

**Funding:**

International Agency for Research on Cancer (IARC/WHO).

**Research in context**

**Evidence before this study**

A search of PubMed conducted on March 1st, 2021, limited to articles in English, but not by date, using the terms “EBV”, “lymphoma”, “prevalence”, and “EBER”, found that no large, unselected series including the whole spectrum of lymphoma subtypes have been systematically tested for the presence of EBV in the tumour tissue. The absence of consensus on a threshold for EBV (Epstein-Barr virus) positivity also limits comparison between studies on specific subtypes and discussion about possible causality.

**Added value of this study**

This study is one of the largest of very few series of lymphoma patients, for which systematic testing for EBV by EBER-ISH (EBV-encoding RNA in situ hybridisation) has been performed. Lyon-Sud University Hospital is a tertiary reference centre for the study and treatment of lymphoma in France, and harbours a strong expertise in the histopathology of lymphomas. We show our results according to different thresholds, using a scoring system designed in accordance with the current state of knowledge in the literature. The comprehensive description of EBV prevalence in lymphomas detailed here could facilitate extrapolation by lymphoma subtype at a national level.

**Implications of all the available evidence**

Extrapolating our results to the large, French Lymphopath database reporting on the distribution of lymphoma subtypes, we estimated that 7·7% of lymphomas (Hodgkin and non-Hodgkin lymphomas) diagnosed in France were strongly associated with, and potentially attributable to EBV (score=3). When we also considered lymphomas attributed a score of 2, where EBV was present but a causal association with EBV still debatable, this fraction reached 9·8%. Our study adds to the growing amount of data in favour of the development of preventive measures (such as vaccines) and therapeutic strategies targeting EBV, or EBV-related cancers.

Alt-text: Unlabelled box

## Introduction

Epstein–Barr virus (EBV), a member of the human herpesvirus family, was first isolated from cultured B-cell lymphoma in 1964.^1^ Found worldwide, it is one of the most common human infectious agents, with an estimated prevalence of over 90%. Transmission is primarily oral. Infection is usually asymptomatic in early childhood and may manifest as infectious mononucleosis in adolescents and young adults. EBV permanently changes the host's resting B-cells into latently infected lymphoblastoid cell lines.^2^ Early studies demonstrated that the virus is a potent lymphotropic agent capable of transforming B-cells in vitro into a state of continuous proliferation called ‘immortalisation’. The presence of clonal EBV episomes detected in virtually all endemic African Burkitt lymphoma (BL) cells suggested that the tumour develops from a single infected cell, and that EBV has a role in the initiation of neoplastic transformation, which led it to be designated as the first human carcinogenic virus.^3^

EBV has been associated with several epithelial cancers (i.e. nasopharyngeal cancer in Eastern Asia and a subset of gastric cancers worldwide) but its precise role in carcinogenesis remains to be determined. The strongest causal associations have been shown for Hodgkin lymphoma (HL) and BL.^2^^,^^4^ In 2018, around 40 000 non-Hodgkin lymphomas (NHL) (50% of new cases) and 6700 BL (55%) were estimated to be attributable to EBV worldwide, with wide regional variations.^5^

Very few studies have systematically tested the entire spectrum of HL and NHL for the presence of EBV, despite mounting evidence for its causal role in specific lymphoma subtypes such as diffuse large B-cell lymphomas (DLBCL) or angioimmunoblastic T-cell lymphomas (AITL).^6^^,^^7^ Similarly, EBV expression in small B-cell lymphomas is suspected to be rare but has seldom been systematically studied. At a time when new techniques to develop vaccines that could target EBV are becoming available, it is important to improve estimates of the burden of lymphoma attributable to this virus that could potentially be averted.

In the present study, we aimed to describe the prevalence of EBV in a large series of patients diagnosed with any type of lymphoma during the year 2020, at the Lyon-Sud University Hospital, France.

## Methods

### Recruitment of patients and case selection

All lymphoma cases registered between Jan 1 and Sept 30, 2020 were retrospectively retrieved from the Pathology Department of the Lyon-Sud University Hospital, a French reference centre for the diagnosis of lymphoid malignancies, in particular T-cell lymphomas^8^^,^^9^ within the Lymphopath network.^10^

Diagnoses were reported according to the 2016 World Health Organization (WHO) classification of Tumours of Haematopoietic and Lymphoid tissue,^11^^,^^12^ and categorised into types and subtypes according to the 2010 update of the InterLymph hierarchical classification of lymphoid neoplasms for epidemiologic research.^13^ Data including age, sex, ICD-O code (WHO International Classification of Diseases for Oncology),^12^ medical history of lymphoma, and anatomic location were collected. Lymphoma cases were included, irrespective of age. Cutaneous lymphomas, indolent lymphoproliferative disorders, and lymphomas or lymphoproliferations occurring in immunosuppressed patients (i.e., with a history of organ transplantation or HIV infection), in particular EBV–associated B-cell lymphoproliferations, were excluded.

Patients who had multiple biopsies to reach diagnosis were included only once. Patients with a known history of the same lymphoma (histologically confirmed) having received one or more treatments were considered as relapsing cases at the time of the study. Patients with DLBCL, with a known history of small B-cell lymphoma (histologically confirmed), or even with a low-grade contingent on the biopsy, were considered as transformed cases if the phenotype was concordant and EBV-associated B-cell lymphoproliferation could be ruled out at diagnosis.

### In situ hybridisation of EBV-encoded RNA

The presence of EBV was assessed in tumour tissue using the in situ hybridisation (ISH) technique targeting EBER (EBV-encoded RNA) -1 or -2, the gold standard for detecting EBV in tumour cells. EBER-ISH testing was performed on deparaffinised tissue sections using a fluorescein isothiocyanate-coupled specific peptidic nucleic acid probe (800-2842; Roche Diagnostics, Ventana Medical Systems, Mannheim, Germany). EBV+ DLBCL-not otherwise specified (NOS), was included as positive control.

### Slide reviewing

Double-blinded assessment of EBER RNA expression in tumour cells was conducted by two haemato-pathology experts (ATG, MD). Discordant cases were reviewed collegially. A scoring system from 0 to 3 was designed for this study (Table 1), in accordance with the current state of knowledge in the literature; “score 1” being presence of scattered EBV bystander cells (<10% cells, defined as latently infected lymphocytes that might be present in any viral carrier);^14^^,^^15^ “score 2” being an intermediate score (≥10% stained cells); and “score 3” meaning “EBV-associated lymphoma”. The score of 3 was assigned only to lymphomas known to be possibly associated with EBV, i.e. DLBCL with ≥80% stained cells^16^^,^^17^ (EBV+ DLBCL-NOS), classical HL (cHL) with stained Hodgkin/Reed Sternberg cells,^18^ and NK and T-cell lymphomas with EBER-positive staining in nearly all neoplastic cells.^19^ The intermediate score (score 2) was used to distinguish between small B-cell lymphomas with scattered by-stander cells (<10%, score 1) from small B-cell lymphomas with a larger number of EBV+ B-cells (>10% EBV+ cells, score 2). Lymphomas for which the association with EBV and the scoring to establish it has not yet been objectively described in the literature (e.g. nodal lymphoma of T follicular helper (TFH) cell origin...) and lymphomas with an insufficient number of EBV+ cells to reach the cut-off accepted in the literature were also included in this category. For example, a DLBCL with 40% EBER+ lymphoma cells was classified as DLBCL-NOS with a score 2, and a DLBCL with >80% EBER+ lymphoma cells was classified as DLCBL-EBV+ with a score 3.

**Table 1:** Description of the scoring system used to assess the strength of the association between EBV presence in tumour tissue (by EBER-ISH) and principal subtypes of lymphomas.

	Score 1 Bystander	Score 2 Potential association with EBV	Score 3 Strong association with EBV
**Diffuse large B-cell lymphoma (DLBCL) and other large B-cell lymphoma**^16^^,^^17^	<10% stained cells	≥10 to <80% stained cells	≥80% of stained cells
**Classical Hodgkin lymphoma (cHL)**^18^	<10% stained cells	≥10% stained cells in the microenvironment, but negative Reed Sternberg cells.	Positive Reed Sternberg cells
**NK and T-cell lymphoma**^19^	<10% stained cells	*Never described*	Nearly all T-cells positive
**Angio-immunoblastic T-cell lymphoma (AITL)**^19^	<10% stained cells	-	Positive large CD30+ B-cells
**Other lymphoma (including follicular lymphoma and other small B-cell lymphomas**	<10% stained cells	≥10% stained cells	*Not applicable*

EBER-ISH=EBV-encoding RNA in situ hybridisation. EBV=Epstein-Barr virus.

The above-mentioned categories of lymphomas, known to be associated with EBV and with a well-defined score to establish this association, were thus classified with a score ranging from 0 to 3. Other lymphomas were classified with a score ranging from 0 to 2.

For AITL, some authors argue that neoplastic T cells in AILT may be EBV-infected. However, most studies showed EBV to be mainly or exclusively present in non-neoplastic bystander B cells.^20^ In the present study, we did not dispose of a double staining CD3/LMP1, and only AITL with positive large CD30+ B-cells were classified as "EBV-associated" (score 3). Score 2 was thus not applicable to this category.

### Compliance with ethical standards in research

The study protocol was approved by the local ethical committee, and all participating patients provided informed consent according to the French legislation (National Commission on Informatics and Liberty n° 21_5700).

### Statistical analysis

EBV prevalence, as percentages, is reported for lymphoma cases successfully tested by EBER-ISH. Comparisons were assessed using Fischer exact test or Wilcoxon rank-sum test, as appropriate. To provide estimates of the frequency of lymphoma associated with EBV at a national level, EBV prevalence data by lymphoma subtype assessed in this study were applied weighted according to the relative distribution of specific lymphoma entities reported in the Lymphopath database including 36 920 lymphomas diagnosed between 2010 and 2013 and reviewed by the Lymphopath network pathologists.^10^ Statistical tests were two-sided, and all analyses were done using STATA version 14.

### Role of the funding source

The funder of the study had no role in study design, data collection, data analysis, data interpretation, writing of the report or decision to submit for publication. The authors had full access to all the data in the study, and they accept responsibility to submit for publication.

## Results

**Figure 1:**
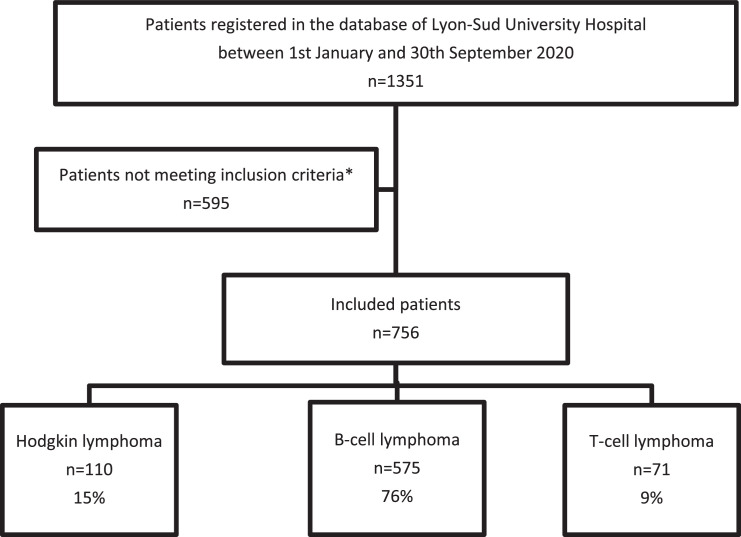
**Flowchart of the study**. *Not meeting criteria: 595 excluded patients, including 536 patients with non-lymphomatous lesions (389 reactional proliferations, 22 indolent lymphoproliferative disorders, and 125 tumours other than lymphomas (i.e., carcinoma, melanoma, sarcomas, myeloid proliferations and benign tumours)); 2 EBV-induced lymphoproliferations occurring in transplanted patients; 24 cases sent for DNA extraction or other analysis, and for whom no histological diagnosis was requested; and 33 unclassifiable lymphomas due to insufficient or damaged tissue.

Among 1351 reviewed cases, 595 were excluded for the following reasons (Figure 1): 536 patients with non-lymphomatous lesions including 389 reactional proliferations, 22 indolent lymphoproliferative disorders, and 125 tumours other than lymphoma (i.e., carcinoma, melanoma, sarcomas, myeloid proliferations or benign tumours); 2 EBV-induced lymphoproliferations occurring in transplanted patients; 24 cases sent for DNA extraction or other analysis, and for whom no histological diagnosis was requested; and 33 unclassifiable lymphomas due to insufficient or damaged tissue.

### Distribution of lymphoma subtypes

A total of 756 eligible lymphoma cases over the study period were included in the final analysis (Table 2). Of the 110 Hodgkin lymphomas (14.6% of total tested), 58% were in men and median age was 38 years (interquartile range (IQR) 29–61). The 575 B-cell lymphomas (76·1%) included 57% men and median age was 71 years (IQR 60–78), whereas in 71 NK and T-cell lymphomas (9·4%), 73% were in men and median age was 67 (49–77). The vast majority (90%) were newly diagnosed cases, not relapse or recurrent cases. Compared to the Lymphopath database,^10^ this series displayed slightly higher proportions of follicular and T-cell lymphomas and a slightly lower proportion of plasma cell neoplasms (Table 2).

**Table 2:** Characteristics of lymphoma patients diagnosed at Lyon-Sud from 1st January to 30th September 2020 and comparison with Lymphopath French network database.

		Study total N (%)	Incident cases^a^ N (%)	Gender (% male–% female)	Age Median (IQR)	Lymphopath^b^ N (%)	p-value^c^
**Hodgkin lymphoma**		110 (14·6)	101 (91·8)	58·2–41·8	38 [29–61]	4713 (12·5)	0·87
**B-cell non-Hodgkin lymphoma (NHL)**		575 (76·1)	506 (87·8)	57·1–42·9	71 [60–78]	25 121 (79·0)	
	Burkitt lymphoma (BL)	3 (0·4)	3 (100)	100–0·0	58 [16–78]	408 (1·3)	0·05
	Follicular lymphoma (FL)	158 (20·9)	129 (81·6)	57·0–43·0	70 [61–77]	5208 (16·4)	0·06
	Other large B-cell NHL^d^	240 (31·7)	231 (96·3)	55·0–45·0	71 [58–79]	10 744 (33·8)	0.74
	Other small B-cell NHL^d^	158 (20·9)	127 (80·4)	60·1–39·9	72 [62–79]	7590 (23·9)	<0·01
	Plasma cell neoplasm (PCN)	16 (2·1)	14 (87·5)	56·3–43·7	76 [63–79]	1171 (3·7)	0·03
**NK and T-cell NHL**		71 (9·4)	65 (91·5)	71·8–28·2	67 [49–77]	1965 (6·2)	
	Extranodal NK/T-cell lymphoma, nasal type (ENKL)	4 (0·5)	2 (50·0)	50·0–50·0	54 [38–63]	123 (0·4)	1·0
	Angioimmunoblastic T-cell lymphoma (AITL)	29 (3·8)	25 (86·2)	75·9–24·1	67 [50–77]	739 (2·3)	0·03
	Other T-cell NHL	38 (5·0)	38 (100)	71·1–28·9	64 [48–77]	1103 (3·5)	<0·01
**Total**		756 (100)	672 (88·9)	58·7–41·3	68 [54–77]	31 799 (100)	

a Non-incident cases are relapse/recurrent.

b Data from the Lymphopath database^10^ after subtracting unclassified lymphomas.

c Two-sided comparison between proportions (Lymphopath versus incident cases in current study).

d Excluding FL. IQR=Interquartile range.

Detailed distribution by sub-type is shown in Table 3. HL were composed of 87 (79·1%) cHL and 23 (20·9%) nodular lymphocyte-predominant HL (NLPHL). Among cHL, there were 50 nodular sclerosis cHL (NSCHL), 5 lymphocyte-rich cHL (LRCHL), 7 mixed-cellularity cHL (MCCHL), 2 lymphocyte-depleted cHL (LDCHL), and 23 NOSCHL. Two NOSCHL represented Richter's syndrome of chronic lymphocytic leukaemia (CLL), and 9 additional cases were relapses of known cHL (6 NOSCHL and 3 NSCHL).

**Table 3:** Frequency of EBV detection using EBER-ISH among subtypes of lymphoma.

				Total	EBER results N (%)				
					N tested	Score 0	Score 1	Score 2	Score 3
**Hodgkin lymphoma (HL)**				**110**	**105**^a^	**67 (64)**	**9 (9)**	**2 (2)**	**27 (26)**
	Classical Hodgkin lymphoma (cHL)			87	87	52 (60)	6 (7)	2 (2)	27 (31)
	*Lymphocyte-rich (LRCHL)*			5	5	4 (80)	0 (0)	0 (0)	1 (20)
	*Mixed-cellularity (MCCHL)*			7	7	4 (57)	0 (0)	0 (0)	3 (43)
	*Lymphocyte-depleted (LDCHL)*			2	2	1 (50)	0 (0)	0 (0)	1 (50)
	*Nodular sclerosis (NSCHL)*			50	50	32 (64)	6 (12)	1 (2)	11 (22)
	*cHL, NOS*			23	23	11 (48)	0 (0)	1 (4)	11 (48)
	Nodular lymphocyte predominant Hodgkin lymphoma (NLPHL)			23	18^a^	15 (83)	3 (17)	0 (0)	0 (0)
**Non-Hodgkin lymphoma (NHL)**				**646**	**511**^a^	**430 (84)**	**39 (9)**	**11 (2)**	**31 (5)**
**B-cell NHL**				**575**	**440**^a^	**395 (90)**	**23 (5)**	**9 (2)**	**13 (3)**
	**Follicular lymphoma (FL)**			**158**	**97**^a^	**79 (90)**	**7 (8)**	**2 (2)**	**0 (0)**
	Grades 1–2			133	72^a^	63 (88)	7 (10)	2 (3)	0 (0)
	Grade 3A			18	9^a^	9 (100)	0 (0)	0 (0)	0 (0)
	Grade 3B			6	6	6 (100)	0 (0)	0 (0)	0 (0)
	Paediatric follicular lymphoma (PFL)			1	1	1 (100)	0 (0)	0 (0)	0 (0)
	**Burkitt lymphoma (BL)**			**3**	**3**	**3 (100)**	**0 (0)**	**0 (0)**	**0 (0)**
	**Other large B-cell lymphoma**								
	Diffuse large B-cell lymphoma (DLBCL)			223	223	206 (92)	2 (1)	4 (1)	11 (5)
	EBV-positive diffuse large B-cell lymphoma, NOS			9	9	0 (0)	0 (0)	0 (0)	9 (100)
	Primary DLBCL of the central nervous system (CNS DLBCL)			16	16	15 (94)	0 (0)	0 (0)	1 (6)
	T-cell/histiocyte-rich large B-cell lymphoma (THRLBCL)			6	6	6 (100)	0 (0)	0 (0)	0 (0)
	Primary mediastinal large B-cell lymphoma (PMBL)			18	18	17 (94)	0 (0)	1 (6)	0 (0)
	Plasmablastic lymphoma (PBL)			3	3	2 (67)	0 (0)	0 (0)	1 (6)
	DLBCL-NOS			171	171	166 (97)	2 (1)	3 (2)	0 (0)
	High-grade B-cell lymphoma (HGBL)			16	16	16 (100)	0 (0)	0 (0)	0 (0)
	Mediastinal grey-zone lymphoma (MGZL)			1	1	1 (100)	0 (0)	0 (0)	0(0)
	Plasma cell neoplasm			16	12^a^	12 (100)	0 (0)	0 (0)	0 (0)
	Plasmacytoma			2	2	2 (100)	0 (0)	0 (0)	0 (0)
	Plasma cell myeloma (PCM)			14	10^a^	10 (100)	0 (0)	0 (0)	0 (0)
	**Other small B-cell lymphoma**								
	Chronic lymphocytic leukaemia (CLL)			31	19^a^	14 (74)	4 (22)	1 (5)	0 (0)
	Mantle cell lymphoma (MCL)			28	20^a^	19 (95)	1 (5)	0 (0)	0 (0)
	Lymphoplasmacytic lymphoma (LPL)			12	9^a^	8 (89)	0 (0)	1 (11)	0 (0)
	Splenic diffuse red pulp small B-cell lymphoma (SRPL)			2	2	1 (50)	1 (50)	0 (0)	0 (0)
	Marginal zone lymphoma (MZL)			85	47^a^	37 (79)	8 (17)	2 (4)	0 (0)
	Mucosae-associated lymphomoid tissues (MALT) lymphoma			41	19^a^	16 (84)	3 (16)	0 (0)	0 (0)
	Nodal marginal zone lymphoma (NMZL)			39	25^a^	18 (72)	5 (20)	2 (8)	0 (0)
	Splenic marginal zone lymphoma (SMZL)			5	3^a^	3 (100)	0 (0)	0 (0)	0 (0)
**NK and T-cell NHL**				**71**	**71**	**35 (49)**	**16 (23)**	**2 (3)**	**18 (25)**
**NK/T-cell NHL**									
	Extranodal NK/T-cell lymphoma, nasal type (ENKL)			4	4	0 (0)	0 (0)	0 (0)	4 (100)
**T-cell NHL**				**67**	**67**	**35 (52)**	**16 (24)**	**2 (3)**	**14 (21)**
	T acute lymphoblastic leukaemia/lymphoma (T-ALL)			3	3	3 (100)	0 (0)	0 (0)	0 (0)
	Anaplastic large cell lymphoma (ALCL)			12	12	10 (83)	2 (17)	0 (0)	0 (0)
	ALK-negative anaplastic large cell lymphoma (ALK(-) ALCL)			11	11	9 (82)	2 (18)	0 (0)	0 (0)
	Breast implant-associated anaplastic large cell lymphoma			1	1	1 (100)	0 (0)	0 (0)	0 (0)
	Angioimmunoblastic T-cell lymphoma and other nodal lymphoma of T follicular helper (TFH) cell origin			33	33	8 (24)	9 (27)	2 (6)	14 (42)
	Angioimmunoblastic T-cell lymphoma (AITL)			29	29	6 (21)	9 (31)	0	14 (48)
	Nodal peripheral T-cell lymphoma with TFH phenotype			3	3	2 (67)	0 (0)	1 (33)	0 (0)
	Follicular T-cell lymphoma			1	1	0 (0)	0 (0)	1 (100)	0 (0)
	Peripheral T-cell lymphoma, rare subtypes			4	4	3 (75)	1 (25)	0 (0)	0 (0)
	Peripheral T-cell lymphoma, NOS			11	11	9 (82)	0 (0)	0 (0)	0 (0)
	Intestinal T-cell lymphoma			4	4	2 (50)	2 (50)	0 (0)	0 (0)
	Enteropathy-associated T-cell lymphoma (EATL)			2	2	1 (50)	1 (50)	0 (0)	0 (0)
	Monomorphic epitheliotropic intestinal T-cell lymphoma (MEITL)			1	1	0 (0)	1 (100)	0 (0)	0 (0)
	Intestinal T-cell lymphoma, NOS			1	1	1 (100)	0 (0)	0 (0)	0 (0)
**Total**				**756**	**616**				

a Not all tested, due to lack of available material. EBER-ISH=EBV-encoding RNA in situ hybridisation. EBV=Epstein Barr virus.

Among NHL, the 158 follicular lymphomas (FL) were composed of 133 grade 1–2, 18 grade 3A, 6 grade 3B, and 1 paediatric FL. Among FL, 29 were considered relapsing cases.

Other B-cell lymphomas were composed of 3 BL, 223 DLBCL, 16 high-grade B-cell lymphomas, 1 B-cell lymphoma unclassifiable with intermediate features between DLBCL and cHL or mediastinal grey zone lymphoma (MGZL), 158 other small B-cell lymphomas and 16 plasma cell neoplasms. Eight DLBCL-NOS and 1 high grade B-cell lymphoma had a history of large B-cell lymphoma and were considered as relapses. Two T-cell/histiocyte-rich large B-cell lymphoma and 23 DLBCL-NOS had a history of low-grade B-cell lymphoma and were considered as transformations. Among small B-cell lymphomas, 31 were relapses (13 CLL, 7 mucosae-associated lymphoid tissue (MALT) lymphomas, 1 lymphoplasmacytic lymphoma (LPL), 9 mantle cell lymphomas (MCL) and 1 splenic marginal zone lymphoma (SMZL)).

The 71 NK and T-cell lymphomas were composed of 4 extranodal NK/T-cell lymphoma, nasal type (ENKL) and 67 T-cell lymphomas, encompassing 3 T-cell acute lymphoblastic leukaemias/lymphomas (T-ALL), 12 anaplastic lymphomas (ALCL), 29 AITL, 4 other nodal lymphomas of T follicular helper (TFH) cell origin, 15 peripheral T-cell lymphomas, and 4 intestinal T-cell lymphomas. Four of the peripheral T-cell lymphomas were specified as rare subtypes and included one nodal gamma/delta T-cell lymphoma, two HTLV-1 associated T-cell leukaemias/lymphomas and one large granular lymphocytic leukaemia. Six of the 71 NK and T-cell lymphoma cases had a history of the same lymphoma and were considered as relapses (2 ENKL, 4 AITL).

### EBER results

According to the 2016 WHO recommendations for classification of lymphoid neoplasms,^12^ EBER-ISH was performed systematically on all types of cHL, large B-cell lymphomas (including FL grade 3B), as well as for all NK and T-cell lymphomas. For other types of lymphomas, when EBER was not performed systematically, we performed the test retrospectively, if adequate material was still available (Table 3). Overall, lymphoma was found to be strongly associated with EBV in 27/87 (31%) cHL, 12/223 (5%) DLBCL, and 18/71 (25%) NK and T-cell lymphomas. There was no significant association between EBV status and age or sex for cHL, B-cell or T-cell lymphoma.

### Hodgkin lymphomas

Among the 105 HL tested, EBV prevalence based on a score of 3 was 25·7% (95% confidence interval (CI) 17·4–34·0) and exclusively concerned subtypes of cHL for which the prevalence was 31% (21·4–40·6) including 11 NSCHL (22%), 1 LRCHL (20%), 3 MCCHL (43%), 1 LDCHL (50%), and 11 NOSCHL (48%). Using a threshold of 2, EBER-stained cells were seen in 1 additional NSCHL and 1 NOSCHL (Figure 1), raising the prevalence to 33·3% (23·5-43·1) among cHL. Bystander cells (score 1) were seen in 6 NSCHL and 3 NLPHL. Two patients had a history of CLL and were therefore classified as a transformation of low-grade lymphomas (Richter syndrome). Of these two patients, only one had EBV+ cells (positive Hodgkin cells, classified as score 3). EBER positivity was significantly associated with relapse using a score ≥2 (*p* = 0·01) or a score of 3 (*p* < 0·05).

### Follicular lymphomas

**Figure 2:**
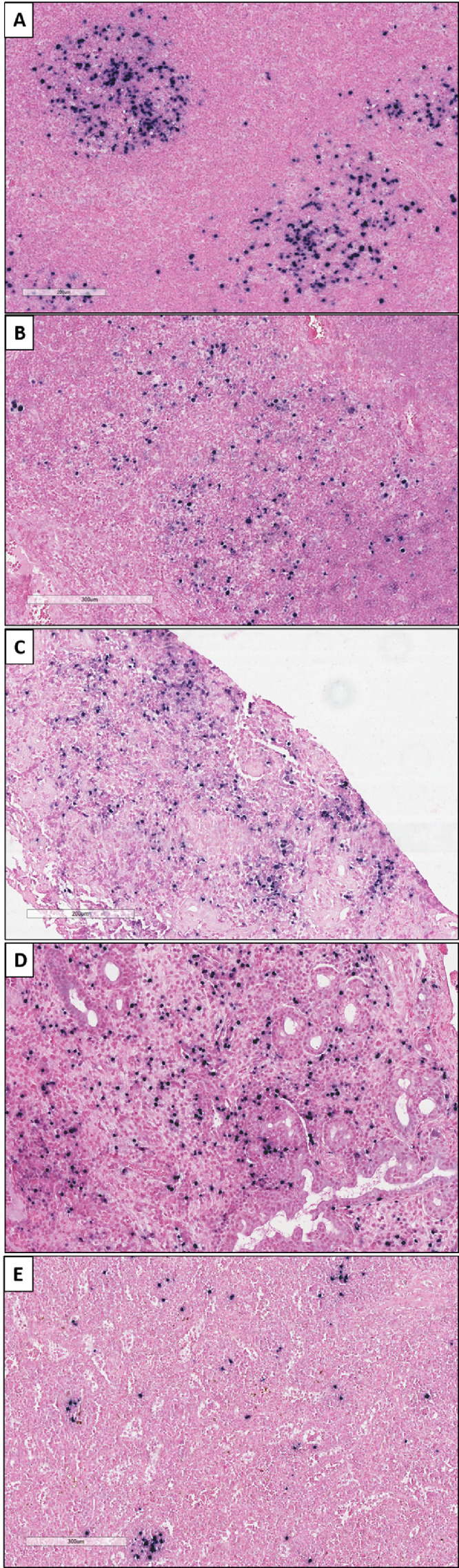
**Positive results of EBER-ISH using a threshold of 2 (≥10% stained cells) in small B-cell lymphomas**. Since small B-cell lymphomas are not accepted as being associated with EBV, a score of 3 was not possible in this category. (A-B) Grades 1–2 Follicular lymphomas (magnification x10); (C) Chronic lymphocytic leukaemia (magnification x10); (D) Lymphoplasmacytic lymphoma (magnification x10). (E) Nodal marginal zone lymphoma (magnification x10). EBER-ISH=EBV-encoding RNA in situ hybridisation. EBV=Epstein-Barr virus.

EBER-ISH testing had been performed at diagnosis on 41/158 (25%) cases of FL. Material was still available for testing in 56 unanalysed FL cases to a total 97/158 (61%) cases. Two FL grade 1–2 (3%, 0–6·4) were positive with a score of 2 (Figure 2A, B). One of them was a relapsing case after chemotherapy. Bystander cells (score 1) were seen in 7/72 (10%) FL grade 1–2 (Table 3). All other FL, including the 6 large FL grade 3B, were negative.

### Burkitt lymphoma and other large B-cell lymphomas

The 3 BL studied were negative for EBV staining (score 0). Among them, none were immunosuppressed, and there were no HIV+ patients. Eleven cases (4·9%, 2·1–7·7) of the 223 DLBCL were assigned a score of 3 with ≥80% positive tumour cells. Of these, 9 were classified as EBV+ DLBCL-NOS, 1 as central nervous system (CNS) DLBCL, and 1 as plasmablastic lymphoma (PBL). Using a score ≥2, EBER-stained cells were seen in 3 additional DLBCL-NOS, and 1 primary mediastinal large B-cell lymphoma (PMBL), for a total of 15 large B-cell lymphoma cases positive for EBV (6·7%, 3·4–9·9) (Table 3).

Bystander cells were seen in 2 DLBCL-NOS. In DLBCLs, there was no statistically significant association between EBER positivity (score ≥2 or score 3) and transformation or relapse. Of note, one of the 9 EBV+ DLBCL-NOS cases was immunosuppressed due to rheumatoid arthritis treated by methotrexate.

In plasma cell neoplasms, EBER-ISH testing was performed in 12/16 identified cases (75%) including both cases of plasmacytoma and 10/14 myelomas (71%). No association with EBV nor the presence of bystander EBV-positive cells was detected (Table 3).

### Other small B-cell lymphomas

EBER-ISH testing had been performed at diagnosis on 57/158 (36%) other small B-cell lymphomas. Material remained available for testing in 40 unanalysed cases to a total 97/158 (61%) cases of small B-cell lymphomas including 19 CLL (61%), 20 MCL (71%), 9 LPL (75%), 2 SRPL (100%), and 47 marginal zone lymphomas (MZL) (55%).

Using a threshold of 2, EBER testing was positive in 1/19 CLL (5%), 1/9 LPL (11%), and 2/47 (MZL (4%), both being nodal MZL (NMZL) (Table 3, Figure 2C, D, E) of which one had a history of traumatic splenectomy. No association was seen between score 2 and relapse. Bystander cells were seen in 5 NMZL, 3 MALT lymphomas, 1 splenic diffuse red pulp small B-cell lymphoma (SRPL), 4 CLL, and 1 MCL. Among them, 3 NMZL, 1 MALT and 1 CLL were relapsing cases.

### NK and T-cell lymphomas

All NK and T-cell lymphomas were tested at diagnosis: 14/29 AITL (48·3%, 30·5–66·1) presented positive large CD30+ B-cells and were classified as "EBV-associated" (score 3) and all 4 ENKL (100%) presented more than 80% of EBV+ cells and were assigned a score of 3. None of the other subtypes of T-cell lymphomas were strongly associated with EBV (score=3). However, 1/3 nodal peripheral T-cell lymphomas with TFH phenotype and the only follicular T-cell lymphoma in our series were assigned a score of 2. Bystander cells were seen in 16 T-cell lymphomas (2 ALK-negative anaplastic large cell lymphoma (ALK(-) ALCL), 9 AITL, 1 enteropathy-associated T-cell lymphoma (EATL), 1 monomorphic epitheliotropic intestinal T-cell lymphoma (MEITL), 2 peripheral T-cell lymphoma (PTCL)-NOS and 1 HTLV1 associated adult T-cell leukaemia/lymphoma). In the NK and T-cell lymphoma group, there was a statistically significant association between EBER positivity (score ≥2 or score 3) and relapse (*p* < 0·01).

### Extrapolation to the Lymphopath database

Based on the scoring system used in this study and the present results, we estimated that 7·7% lymphomas diagnosed in France were strongly associated with EBV (score 3). If we also consider lymphomas with a score of 2, this fraction reaches 9·8%.

## Discussion

In a series of patients diagnosed in 2020 at the Lyon-Sud University Hospital, France, when using a threshold score of 3 (i.e., a strong, EBV associated lymphoma), lymphoma was found to be associated with EBV in 27/87 (31%) cHL, 12/223 (5%) DLBCL, and 18/71 (25%) NK and T-cell lymphomas, including all 4 cases of ENKL, and 14/29 AITL (48%). This subtype distribution is consistent with Lymphopath^10^ who reported in 2017 15% HL, 79% B-cell NHL, and 6% T-cell NHL. When we consider only incidental cases, our findings report 15% HL, 75% B-cell NHL, and 10% T-cell NHL, in line with other studies.^21^ There may have been some recruitment bias in favour of NK and T-cell lymphomas, including AITL, compared with the Lymphopath database, as Lyon-Sud University Hospital is a French reference centre for T-cell lymphomas.^8^^,^^9^^,^^17^ (Table 2).

We found around one third cHL to be associated with EBV in agreement with Lee et al., 2014, who reported 35% (95% CI 31·8–39·4) EBV-associated cHL in Europe, and similar proportions in North America (32%) and Australia (29%).^22^ Despite the low number of cases with a diagnosed cHL subtype, our data also suggest, in agreement with the literature, that more EBV-positive tumours are found in mixed-cellularity and lymphocyte-depleted cases, and less are found in nodular sclerosis cases.^22^ It should be noted that EBV-positive cases are described more frequently in cHL in Asia, Latin America, and Africa, with proportions ranging from 50% to 74%.^22^

Only 2 FL grade 1–2 (3%, 0–6·4) of the 97 FL examined were EBV-positive (≥10% stained cells), and one of them was a relapse after chemotherapy with no histological evidence of progression or transformation. The largest series estimating the prevalence of EBV-associated FL in unselected cases^23^ defined EBV-positive as cases with ≥10 EBER-positive tumour cell nuclei per 0·5 cm^2^. Mackrides et al. analysed 382 FL and found 10 EBV-positive cases (2·6%, 1·3–4·0). Among them, 7 progressed to higher-grade FL or DLBCL. Based on these observations, they hypothesised that EBV infection may not be the driving event for EBV-positive FL but may act as a facilitator of disease progression.^23^ In the present study, all cases of FL grade 3B (6 cases) and 7 cases of formerly FL subsequently transformed into highly aggressive lymphomas (hence included in the DLBCL-NOS category) were negative for EBER-ISH.

All three BL cases in the present study were negative by EBER-ISH and only 20 to 30% of sporadic BL were previously found to be EBV-associated.^24^ Among DLBCL, the prevalence of EBV+ DLBCL-NOS was low (9/223, 4%), as described elsewhere.^17^ Other DLBCL with ≥80% stained cells (score 3) were 1 CNS-DLBCL and 1 PBL, raising the prevalence of EBV in all DLBCL to nearly 5%. When also considering DLBCL with ≥10 to <80% stained cells (score 2), therefore including 3 DLBCL-NOS and 1 PMBL, the prevalence of EBV was close to 7%. Previous studies have shown CNS-DLBCL to sometimes express EBER RNA,^25^^,^^26^ but not PMBL, which are described as almost always negative for EBV. Therefore, to ensure correct diagnosis of the PMBL case with score 2, we confirmed its primary mediastinal nature by next-generation sequencing. In this study, the presence of a few EBV+ cells in one case of PMBL (10-20% of positive cells) was not expected and difficult to interpret. Nevertheless, this diagnosis was retained after collegial review, taking into account a favourable morphological aspect (fibrous background, "Hodgkin-like" cells), a typical immunohistochemical phenotype (CD20+ CD23+ CD30+ PDL1+), and a characteristic NGS profile (pathogenic variants of RHOA, TNFAIP3, SOCS1, GNA13, ITPKB and BTG1), as described by Sarkory and al.^26^ A few similar PMBL cases with EBV+ cells have been published in the literature.^27^ Since this PMBL/EBV association is not very well described, this case was classified as an intermediate score (score 2), and not as a score 3 (EBV-associated lymphoma).

None of the 16 plasma cell neoplasms seen in our series was associated with EBV, concordant with a recent case series of 147 immunocompetent patients, all EBV-negative.^28^

Regarding the disparate group of other small B-cell lymphomas, EBER-ISH testing had been performed at diagnosis in 73 among the 309 cases. Testing for an association with EBV is not routinely recommended in small cell B lymphomas but may be performed in certain situations, such as: differential diagnosis between T-cell lymphoma and MZL with TFH hyperplasia, a high number of large B-cells, an immunocompromised patient, or relapse after chemotherapy. Among the cases not tested at diagnosis, we were able to test 96 additional cases retrospectively. We found rare cases with ≥10% stained cells (score 2), namely in 1 CLL, 1 LPL, and 2 NMZL. The significance of score 2 in these subtypes, not known to be associated with EBV, is debatable and may vary for each subtype. For NMZL, the presence of ≥10% EBV-positive cells could indicate a causal role for EBV, as similar cases have been described in immunodeficiency.^29^ Of note, one NMZL in our series had a history of traumatic splenectomy.

Of the 71 NK and T-cell lymphomas included in our series, all 4 cases of ENKL, and 14/29 AITL (48%) were EBV-positive with a score of 3. Bystander EBV cells were also observed in half of the NK and T-cell lymphoma group, including in 6 additional AITL. How EBV is involved in the occurrence of AITL is still controversial.^7^^,^^30^ While the T neoplastic cells are usually EBV-negative, the virus is frequently present in surrounding B-cells, in variable proportions.^19^ A score of ≥2 in our series was significantly associated with relapse, consistent with other studies showing that EBV may play a negative role in the pathogenesis and prognosis of this lymphoma.^7^^,^^31^ In ENKL, EBV is observed in almost all tumour cells with a frequent type II latency pattern, suggesting that the virus is involved in the early stages of lymphomagenesis. Moreover, elevated EBV DNA loads are correlated with poor prognosis.^32^

Our work has several strengths. This study is one of the largest of very few series of lymphoma patients for whom systematic testing for EBV by EBER-ISH has been performed. Our findings are consistent with the literature, although previous studies (with the exception of a recent study in Rwanda)^33^ generally focused on one lymphoma subtype not attempting to cover the whole spectrum of subtypes. Lyon-Sud University Hospital is a tertiary reference centre for the study and treatment of lymphoma in France and harbours a strong expertise in the histopathology of lymphomas. While the distribution of lymphomas in this series might be biased toward a slight excess of T-cell lymphoma compared to other centres or to the national Lymphopath database, the comprehensive description of EBV prevalence in lymphomas detailed here could facilitate extrapolation by lymphoma subtype at a national level.

Some limitations should also be mentioned. First, the scoring system used in this work follows the latest WHO recommendations on DLBCL, or expert consensus, but has not been universally adopted.^14^, ^15^, ^16^^,^^18^^,^^19^ The absence of consensus on the best scoring system limits comparisons with work published by other teams. To compensate for this choice, we also gave the results for the intermediate score of 2 and for bystander positive cells (score 1). Second, while most EBER-ISH was done at diagnosis, some testing (e.g. on small B-cell subtypes or FL) was retrospective and was limited by the quality and amount of tumour tissue available. We do not believe, however, that this limitation introduced any kind of bias. Another limitation of this study is that, by design, it excluded de facto all patients with a leukaemic presentation, which might also be associated with EBV. While this design does not affect comparisons with the Lymphopath database, which uses the same inclusion criteria,^10^ our series is therefore not strictly speaking representative of the full spectrum of lymphoma and lymphoid leukaemia as described in the latest WHO classification.^12^

In conclusion, this large, unselected French case series provides strong data on EBV-associated malignant lymphomas and adds to the effort required to obtain a robust estimation of the global burden of cancer attributable to EBV. Based on the Lymphopath distribution of lymphoma subtypes, we estimated that 7·7% of lymphoma (HL and NHL) diagnosed in France were strongly associated with EBV (score 3). When we also considered lymphomas with a score of 2, where EBV is present, but a causal association with EBV is still debatable, this fraction reached 9·8%. Important research remains to be done to demonstrate a causal association between EBV infection and lymphomagenesis, in particular in low-grade lymphomas. However, by expanding the spectrum of EBV-related cancers to other subtypes, such as a fraction of DLBCL and AITL, this study adds to the growing amount of data in favour of the development of preventive measures (such as vaccines) and therapeutic strategies targeting EBV, or EBV-related cancers.^34^

## Contributors

M.D., M.B., A.T.G. and C.D.M. designed the study protocol. All authors actively contributed to study design. M.D. and M.B. collected the data. M.B. and J.D.C. performed the statistical analyses and made the tables and figures. M.B. and J.D.C. have accessed and verified all reported data. M.D., J.D.C., and C.D.M. wrote the paper. All authors contributed to reviewing the manuscript, have approved the final manuscript, had full access to all the data in the study, and accept responsibility to submit for publication.

## Data sharing statement

All data generated or analysed during this study are included in this published article.

## Declaration of interests

PS has received personal fees from Incyte, Chugai, Kite, Novartis and Janssen, and support for attending meetings from Janssen and Kite Gilead. The other authors declare no competing interests.
